# Poly(lactic acid) (PLA)/Poly(butylene succinate-co-adipate) (PBSA) Compatibilized Binary Biobased Blends: Melt Fluidity, Morphological, Thermo-Mechanical and Micromechanical Analysis

**DOI:** 10.3390/polym13020218

**Published:** 2021-01-09

**Authors:** Laura Aliotta, Alessandro Vannozzi, Ilaria Canesi, Patrizia Cinelli, Maria-Beatrice Coltelli, Andrea Lazzeri

**Affiliations:** 1 Department of Civil and Industrial Engineering, University of Pisa, 56122 Pisa, Italy; alessandrovannozzi91@hotmail.it (A.V.); patrizia.cinelli@unipi.it (P.C.); andrea.lazzeri@unipi.it (A.L.); 2 National Interuniversity Consortium of Materials Science and Technology (INSTM), 50121 Florence, Italy; 3 Planet Bioplastics s.r.l., Via San Giovanni Bosco 23, 56127 Pisa, Italy; ilariacanesi@planetbioplastics.com

**Keywords:** poly(lactic) acid (PLA), poly(butylene succinate-co-adipate) (PBSA), binary blends, micromechanical analysis, mechanical tests

## Abstract

In this work poly(lactic) acid (PLA)/poly(butylene succinate-co-adipate) (PBSA) biobased binary blends were investigated. PLA/PBSA mixtures with different compositions of PBSA (from 15 up to 40 wt.%) were produced by twin screw-extrusion. A first screening study was performed on these blends that were characterized from the melt fluidity, morphological and thermo-mechanical point of view. Starting from the obtained results, the effect of an epoxy oligomer (EO) (added at 2 wt.%) was further investigated. In this case a novel approach was introduced studying the micromechanical deformation processes by dilatometric uniaxial tensile tests, carried out with a videoextensometer. The characterization was then completed adopting the elasto-plastic fracture approach, by the measurement of the capability of the selected blends to absorb energy at a slow rate. The obtained results showed that EO acts as a good compatibilizer, improving the compatibility of the rubber phase into the PLA matrix. Dilatometric results showed different micromechanical responses for the 80–20 and 60–40 blends (probably linked to the different morphology). The 80–20 showed a cavitational behavior while the 60–40 a deviatoric one. It has been observed that while the addition of EO does not alter the micromechanical response of the 60–40 blend, it profoundly changes the response of the 80–20, that passed to a deviatoric behavior with the EO addition.

## 1. Introduction

Plastic materials are used constantly in everyday life thanks to their versatility, low cost and huge range of properties. Consequently, the increment of nondegradable plastic waste has remarkably increased so that 150 million tons of plastic per year are consumed worldwide. This fact, combined with the threat of oil depletion, has led in the last decades to the development of biodegradable plastics based on renewable and nonrenewable resources. In fact, the use in specific applications of biodegradable plastics can limit the environmental problems correlated to plastic disposal [1,2,3].

Nowadays different biodegradable polymers are commercially available on the market. Among them poly(lactic) acid (PLA) is the most interesting, due to its low production cost (compared to other biodegradable polymers), good mechanical properties and easy processability (PLA can be manufactured in conventional extrusion, injection molding, blown film extrusion, cast extrusion, thermoforming and three-dimensional (3D)-printing) [4,5,6,7]. However, some PLA drawbacks, such as its brittleness, low toughness and poor heat resistance when employed at a temperature above its glass transition temperature, must be improved to extend its applications [8].

The improvement of PLA toughness (higher ductility and impact resistance) is thus fundamental and for this purpose the simplest approach is physical blending with a more ductile polymer. Among the more investigated biodegradable polymers, those that show a good starting ductility and that can be easily blended with PLA (mainly to increase its toughness) are: poly(butylene succinate) (PBS) [9,10,11], poly(butylene succinate co-adipate) (PBSA) [8,12,13], poly(caprolactone) (PCL) [14,15,16], poly(butylene adipate-co-terephthalate) (PBAT) [17,18,19,20]. Among these possible combinations of biodegradable blends, in this paper the attention has been focused on blending PLA with PBSA. In fact, depending on end-of-life (EOL) options, PBSA possesses a better eco-efficiency compared with the other biopolymers before mentioned [21]; moreover, its availability is very high thanks to the production capacity of around 100,000 tons per year [22]. PBSA is produced by a polycondensation reaction of 1,4-butanediol with succinic acid and adipic acid, that produces a completely aliphatic polyester having high flexibility, excellent impact strength, as well as thermal and chemical resistance and good biodegradability [11,23]. The use of PBSA alone is impracticable in rigid items due to its low stiffness, strength and melting point, but thanks to its low glass transition temperature its behavior is quite similar to a rubber and therefore lends itself very well to physical blending with PLA in order to increase its toughness by the well-known rubber toughening mechanism [20,24,25]. Thus, several research groups recently evidenced the possibility of modulating PLA brittleness by blending with PBSA [26,27,28,29,30,31,32,33].

However, most polymer blends are immiscible due to an unfavorable enthalpy mixing and consequently they form separated phases [34,35,36]. The morphology evolution of a biphasic system depends on the blend composition, processing conditions, rheological properties and interfacial tension of the two constituents [37,38,39,40]. Different morphologies (droplets, co-continuous, double emulsion) can be achieved by tailoring the ratio of PLA with respect to the rubbery polymer and consequently, it is possible to control the mechanical performance of the final material [41,42,43].

- Regarding binary blends, the compatibility/miscibility issues must be considered. It is noteworthy that the introduction of chain extenders that are able to reconnect cleaved chains, increases the molecular weight (consequently increasing the melt strength) [17,44,45]. Different types of chain extenders, also available on the market, have been extensively investigated and reported in literature such as: multi-functional epoxides [46], diisocyanate compounds [47], dianhydride [48], bis-oxazolines, tris(nonyl-phenyl) and phosphate (TNPP) [49]. The introduction of chain extenders also provides a better control of the polymer degradation [17,19,50,51,52] during the process and at the same time enhances the extrusion and injection foamability [52,53]. Moreover, the use of chain extenders can also improve the compatibility between the two phases constituting a binary blend because, especially in the interfacial region, the chain extender can react with both the polymers resulting in the formation of block copolymers acting as effective in situ generated compatibilizers. Chain extenders containing epoxy groups are the most suitable in this case, in fact they are able to react opening the epoxy group ring and creating covalent bonds [45] with both the hydroxyl and carboxylic groups of the polyester chain-ends. The high number of epoxy groups per macromolecule grants efficiency in limiting the decrease of viscosity during processing typical of polyesters that are generally affected by hydrolysis due to atmospheric humidity. For this reason, a multifunctional epoxy oligomer (EO) consisting of styrene, acrylic and glycidyl acrylate units, has been chosen for the binary PLA/PBSA polyester blends. Al-Itry et al. [52] and Wang et al. [54] studied the positive compatibilization effect of a similar EO in a PLA/PBAT system. Lascano et al. [55] explained that EO addition can be advantageous also in PLA/PBSA binary blends (thus very similar to the ones studied in this paper) because it reacts either with the hydroxyl terminal groups of PLA and PBSA, leading to a compatibilization effect and an effective toughening. However, the investigated blends contained up to 30% of PBSA and were not investigated in terms of their failure mechanism and melt fluidity.

In this study a systematic work was carried out by twin screw extruder producing PLA/PBSA blends with different compositions up to 40% of PBSA. These blends were characterized from morphological, melt fluidity, thermal and mechanical points of view. On the basis of the results obtained, the blends showing the best toughness improvement (elongation at break and impact resistance enhancement) were selected and the effect of EO (added at 2 wt.%) as compatibilizer was further investigated. In the second part of the work the micromechanical analysis (which represents a novelty in the study of PLA/PBSA blends) was performed with the help of an optical extensometer, capable of registering both axial and transversal elongation during the tensile tests. In this way it was possible to record the volume variation and to correlate the volume increment to the micromechanical deformation processes (debonding, cavitation, voids growth, etc.). The study was completed with the measurement of the capability of the selected best blends to absorb energy at a slow rate; this measurement was carried out by adopting the elasto-plastic fracture approach based on the ESIS load separation criterion.

## 2. Materials and Methods 

### 2.1. Materials

The materials used in this work are listed below:

- Poly(lactic) acid (PLA) Luminy LX175 produced by Total Corbion PLA. This biodegradable PLA is derived from natural resources, appears as white pellets and contains about 4% of D-lactic acid units. It is a general-purpose extrusion grade PLA that can be used alone or to produce formulated blends or composites; it can be easily processed on conventional equipment for film extrusion, thermoforming or fiber spinning (density: 1.24 g/cm^3^; melt flow index (MFI) (210 °C/2.16 kg): 6 g/10 min).
- Poly(butylene succinate-co-adipate) (PBSA), trade name BioPBS FD92PM, purchased from Mitsubishi Chemical Corporation, is a copolymer of succinic acid, adipic acid and butandiol. It is a soft and flexible semicrystalline polyester suitable for both blown and cast film extrusion (density of 1.24 g/cm^3^; MFI (190 °C, 2.16 kg): 4 g/10 min).
- The epoxy oligomer (EO) used in the work is Joncryl ADR 4468 produced by BASF. It is an oligomeric chain extender having about 20 epoxy groups per macromolecule that reacts with the terminal groups of polycondensates, increasing the melt viscosity (Mw: 7250; density: 1.08 g/cm^3^; epoxy equivalent weight: 310 g/mol).

EO appears as solid flakes that can be easily fed into the lateral feeder of the extruder. It was added to the PLA/PBSA formulations that showed the best toughness and flexibility.

### 2.2. Blends and Sample Preparation

Different blends containing increasing amounts of PBSA (from 5 wt.% to 40 wt.%), were produced in pellets according to the compositions reported in Table 1. To produce the granules, a semi-industrial Comac EBC 25HT (L/D = 44) (Comac, Cerro Maggiore, Italy) twin screw extruder was used. Before extrusion all solid materials were dried in a Piovan DP 604–615 dryer (Piovan S.p.A., Verona, Italy). PLA granules were introduced into the main extruder feeder, while PBSA was fed with a specific lateral feeder. In fact, after setting the weight percentage to be added, this feeder allows to a constant concentration in the melt during the extrusion to be obtained. EO was added only in the formulations that showed enhanced mechanical properties and it was fed separately with another lateral feeder. A fixed quantity of EO equal to 2 wt.% was chosen on the basis of literature works [17,54,56,57,58,59] but also considering the minimum quantity settable in the extruder that guaranteed a constant feeding without dosage problems. The blends containing EO are indicated in brackets and followed by the letter J in Table 1.

The temperature profile of the extruder (11 zones) used for blends preparation was: 150/175/180/180/180/185/185/185/185/185/190 °C, with the die zone at 190 °C for the blends containing up to 20 wt% of PBSA. Due to the major quantity of PBSA (having a lower melting temperature than PLA) decreasing the viscosity, for the blends containing 40 wt.% of PBSA a slightly decrease of 5 °C to the entire temperature profile was carried out; for the 60–40 and (60–40)J blends the temperatures in the zones from 1 to 11 were: 150/170/175/175/175/180/180/180/180/180/185 °C, with the die zone at 185 °C. The screw rate was 300 rpm with a total mass flow rate of 20 kg/h.

The extruded strands were cooled in a water bath at room temperature and reduced in pellets by an automatic knife cutter. All pellets were finally dried again at 60 °C.

After the extrusion, the pellets of the different blends were injection molded using a Megatech H10/18 injection molding machine (TECNICA DUEBI s.r.l., Fabriano, Italy) to obtain two types of specimens: dogbone specimens for tensile tests according to ISO 527-1A (width: 10 mm, thickness: 4 mm, length: 80 mm) and parallelepiped Charpy specimens for Charpy impact test according to ISO 179 (width: 10 mm, thickness: 4 mm, length 80 mm). After the injection molding the Charpy specimens were V-notched in the middle by a V-notch manual cutter (V-notch: 2 mm at 45°).

The samples injection molding parameters are reported in Table 2.

The injection molding was performed using the same temperature profile for all blends. An increase in the cooling time was necessary with the increase of the PBSA content. The mold temperature was also lowered progressively with the increasing amount of PBS from 70 °C for pure PLA to 55 °C for 80–20 and 60–40 blends.

The addition of EO produces a significant increment of the melt viscosity, which in turn raises the pressure requested for filling completely the mold. Consequently, for the blends containing EO the increased viscosity was balanced by increasing both the mold temperature and the injection pressure.

### 2.3. Torque and Melt Flow Rate Analysis

The torque analysis is useful to obtain an indirect estimation of viscosity variations during the extrusion. Torque values were obtained by introducing about 6 g of the extruded granules in a MiniLab II twin-screw mini-compounder (HAAKE, Vreden, Germany). This equipment is able to compound the molten material and at the same time it records the torque values during the extrusion. The extrusion (performed at 190 °C and 100 rpm) was monitored for 1 min and every 10 s an assessment of the torque value was recorded. The measurements were carried out three times and the average value was reported.

The melt flow behavior of the blends was also investigated using a Melt Flow Tester M20 (CEAST, Torino, Italy) equipped with an encoder. The encoder, following the movement of the piston, is able to acquire the melt volume rate (MVR) of the polymer blends. Before the test, granules of the blends obtained from the extrusion process were dried in a ventilated oven (set at 60 °C) for one day. The melt flow rate (MFR), defined as the weight of the molten polymer passing through a capillary (having a specific length and diameter) in 10 min under a specific weight (according the ISO 1133:2005), was also recorded. In particular, the standard ISO 1133D custom TTT was used with a customized procedure: the sample was preheated without the weight for 40 s at 190 °C, then the weight of 2.160 kg was released on the piston and after 5 s a blade cut the spindle starting the real test. At this point the MVR was recorded, every 3 s, by the encoder. All the MVR data were reported with their standard deviation thanks to the CEAST Visuamelt software of the equipment. The MFR values’ standard deviations were calculated by considering the results obtained by the measurements.

### 2.4. Mechanical Characterization

Both tensile and dilatometry tests were carried out on ISO 527-1A dog-bone specimens using an MTS Criterion model 43 universal tensile (MTS Systems Corporation, Eden Prairie, MN, USA) testing machine, at a crosshead speed of 10 mm/min, equipped with a 10 kN load cell and interfaced with a computer running MTS Elite Software. Tests were conducted 3 days after the injection molding process and during this time the specimens were stored in a dry keeper (SANPLATEC Corp., Osaka, Japan) at controlled atmosphere (room temperature and 50% humidity).

For tensile tests at least ten specimens were tested and for each blend composition the average values of the main mechanical properties were reported.

Regarding dilatometry, because of the large number of formulated blends, the tests were carried out only for two selected compositions. At least five specimens for each selected material were tested at room temperature and also in this case the tests were carried out after 3 days from the injection molding process. Transversal and axial specimen elongations were recorded, during the tensile test, using a video extensometer (GenieHM1024 Teledyne DALSA camera) interfaced with a computer running ProVis software (Fundamental Video Extensometer). Furthermore, the data in real-time were transferred to MTS Elite software in order to measure not only the axial and transversal strains but also the load value.

The volume strain was calculated, assuming equal the two lateral strain components, according to the following Equation [60,61,62]:

(1) $$\frac{\Delta V}{V_{0}}=\left(1+\varepsilon_{1}\right){\left(1+\varepsilon_{2}\right)}^{2}-1$$

where the volume variation is Δ*V*, the starting volume is *V*_0_, *ε*_1_ is the axial (or longitudinal) strain and *ε*_2_ is the lateral strain.

The impact tests were performed using V-notched ISO 179 parallelepiped specimens on a Instron CEAST 9050 machine (INSTRON, Canton, MA, USA) equipped with a 15 J Charpy pendulum. At least ten specimens for each blend were tested at room temperature. The impact tests were also carried out 3 days after the injection molding process.

To evaluate the energy stored by the sample before the fracture, three-point bending tests were also carried out. In this case, the tests were performed only on the most significant formulations. The already cited MTS universal testing machine was used. The methodology adopted to calculate the fracture energy at the starting point of crack propagation (*J_Ilim_*) follows the ESIS TC4 load separation protocol [63,64]. According to this protocol, the tests must be carried out at 1 mm/min crosshead speed on 80 mm × 10 mm × 4 mm SENB specimens (that is the same parallelepiped specimen typology adopted for Charpy impact test) cut in two different ways: “sharp” (half notched samples) and “blunt” (drilled in the center with a 2 mm diameter hole and then cut for half width). To obtain the sharp notch (5 mm), during the cutting process, compressed air was used in order to avoid the “notch closing” phenomenon caused by excessive overheating generated by the cutter. A “sacrificial specimen” placed under the “good one” was used to guarantee a correct notch of the sample without closure (qualitatively evaluated with a “passing” paper) and to avoid plastic deformation around it. At least five specimens were tested for each selected blend. Thus the *J_lim_* was calculated following the Load Separation Criterion procedure [65,66,67,68,69] for which it is necessary to construct a load separation parameter curve, obtained from the load (named *P*) vs. displacement (named *u*) during the three-point bending tests. The load vs. displacement curves must be obtained for both types of specimens used (sharp and blunt). In fact, in the sharp specimens the fracture is able to propagate, while in the blunt specimens crack growth cannot occur. At this point it must be defined the *S_sb_* curve that represents the variation of the load separation parameter and is defined as follows:

(2) $$S_{sb}=\frac{P_{s}}{P_{b}}|u_{pl}$$

where the subscripts *s* and *b* indicate the sharp and the blunt notched specimens, respectively. The plastic displacement is denoted as *u_pl_* and it is expressed as:

(3) $$u_{pl}=u-P\cdot C_{0}$$

in which *u* is the total displacement and *C*_0_ is the initial elastic specimen compliance. It must be pointed out that for ductile polymers (like those investigated in this paper), fracture initiation is a complex and progressive process that is characterized by the slow development of the crack front across the thickness of fracture transition [20,67,69]. This limit point is the pseudo-initiation of fracture. Thus, once that limit point was defined, the corresponding *J_lim_* can be calculated as:

(4) $$J_{lim}=\frac{2\cdot U_{lim}}{b\cdot \left(w-a_{0}\right)}$$

where *U_lim_* is the elastic behavior limit point, *b* is the sample thickness, *w* is the sample width and *a*_0_ is the initial crack length.

### 2.5. Optical Analysis

Morphological analysis was carried out on cryo-fractured Charpy samples by FEI Quanta 450 FEG scanning electron microscope (SEM) (Thermo Fisher Scientific, Waltham, MA, USA). To avoid charge build up, the sample surfaces were sputtered (on a LEICA EM ACE 600 High Vacuum Sputter Coater, Wetzlar, Germany) with a thin surface layer of platinum. Image-J software was used to analyze the SEM images and to calculate the number average radius (*R_n_*), the volume average radius (*R_v_*) and the size distribution (*SD*) of the dispersed phase droplets. At least 150 droplets were counted to calculate *R_v_, R_n_* and *SD* parameters according the following Equations [70]:

(5) $$R_{n}=\frac{\sum_{i}n_{i}R_{i}}{n_{i}}$$

(6) $$R_{v}=\frac{\sum_{i}n_{i}R_{i}^{4}}{n_{i}R_{i}^{3}}$$

(7) $$SD=\;\frac{R_{v}}{R_{n}}$$

The fracture surface of the tensile specimen broken during the dilatometric tests offered reliable information about the micromechanical deformations that occurred during the tensile tests. Consequently, some specimens were cold fractured along the tensile direction. In this case the specimens were coated with a thin layer of platinum prior to microscopy to avoid charge build up.

### 2.6. Thermal Characterization

Thermal properties of PLA and PLA/PBSA blends were investigated by calorimetric analysis (Q200 TA- DSC). Nitrogen, set at 50 mL/min, was used as purge gas for all measurements. Indium was used as a standard for temperature and enthalpy calibration of DSC. The materials used for DSC analysis have been cut from the ISO 5271-A dog-bone injection mold specimens. In order to evaluate if an eventual crystallization occurred during the specimen injection molding (affecting the mechanical behavior of the materials), the thermal properties were evaluated considering only the first DSC heating run. The sampling was carried out taking the material in the same region of the specimens to avoid differences ascribable to different cooling rates in the specimen thickness. The samples, with mass between 11.5 and 15 mg, were sealed inside aluminum pans before measurement. PBS granules were also analyzed in order to better understand how its thermal properties could affect the thermograms of the binary blends.

The samples were quickly cooled from room temperature to −50 °C and kept at this temperature for 1 min. Then the samples were heated at 10 °C/min to 200 °C to delete the thermal history then a second cooling scan from −70 °C to 190 °C, at 10 °C/min, was carried out. Melting temperature (T_m_) and cold crystallization temperature (T_cc_) of the blends were recorded at the maximum of the melting peak and at the minimum of the cold crystallization peak, respectively. The enthalpies of melting and cold crystallization were determined from the corresponding peak areas in the thermograms. Where possible the PLA and PBSA crystallinity were calculated according the following Equation:

(8) $$X_{cc,\;PLA\left(or\;PBSA\right)\;}=\frac{\Delta H_{m,\;PLA\;\left(or\;PBSA\right)\;}-\Delta H_{cc,\;PLA\;\left(or\;PBSA\right)\;}}{\Delta H{{}^{\circ}}_{m,\;PLA\;\left(or\;PBSA\right)\;}\cdot \;wt.\;\%\;PLA\;\left(or\;PBSA\right)}$$

where *X_cc_*, is the crystallinity fraction of PLA or PBSA, Δ*H_m_* and Δ*H_cc_* are the melting and cold crystallization enthalpies respectively, while Δ*H°_m_* is the theoretical melting heat of 100% crystalline polymer. For PLA a Δ*H°_m_* value of 93 J/g [71] and for PBSA a Δ*H°_m_* value of 142 J/g were considered [8].

The heat deflection temperature or heat distortion temperature (HDT) corresponds to the temperature at which the polymeric material deforms under a specified load. This property is fundamental during the design and production of thermoplastic components. The HDT is also strictly correlated to the polymer crystallinity, in fact it is noteworthy that a highly crystalline polymer has an HDT value higher than its amorphous counterpart [72]. For this purpose, the determination of the deflection temperature under load (HDT) was carried out on a CEAST HV 3 (INSTRON, Canton, MA, USA) in accordance with ISO 75 (method A). A flexural stress of 1.81 MPa and a bath heating rate of 120 °C/h were used. The sample size was 80 mm × 10 mm × 4 mm. When the sample bar deflects by 0.34 mm, the corresponding bath temperature represents the HDT (Type A) value. At least five measurements were carried out and the average value was reported.

## 3. Results and Discussions

### 3.1. Melt Fluidity, Morphology and Thermal Properties of PLA/PBSA Blends

**Figure 1:**
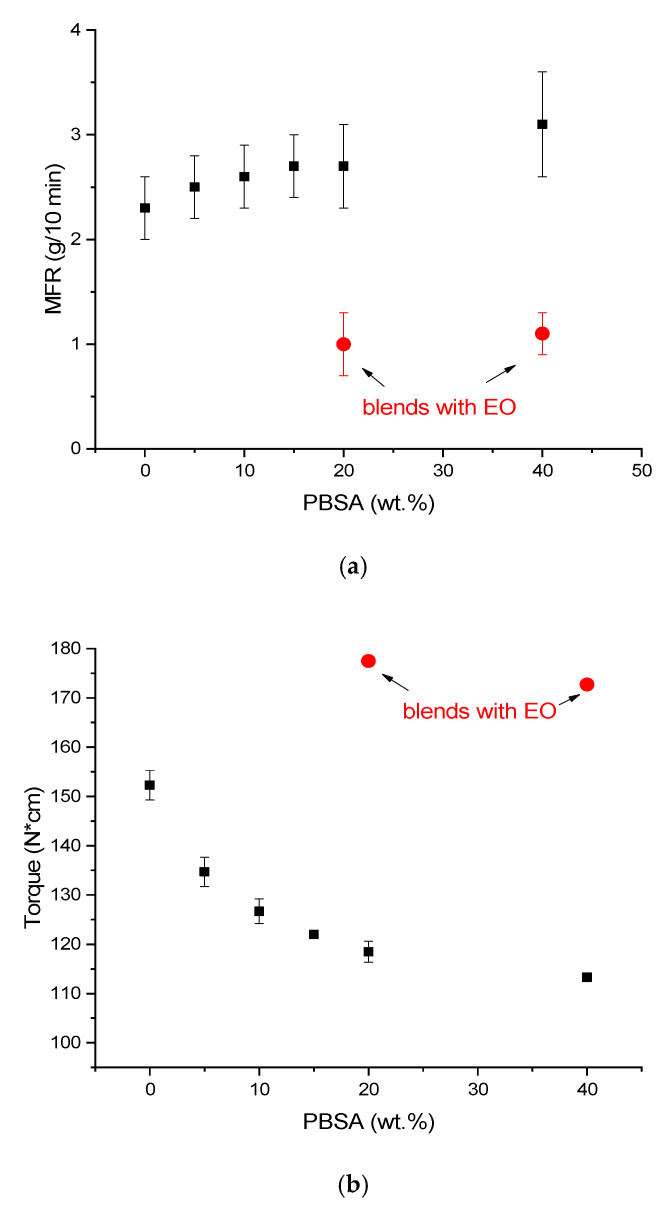
(**a**) Trend of MFR as a function of PBSA content; (**b**) trend of torque as a function of PBSA content.

From the MFR and torque results, reported in Figure 1, it can be observed that the increase of PBSA content resulted in a decrease in torque values and an increment in MFR.

**Figure 2:**
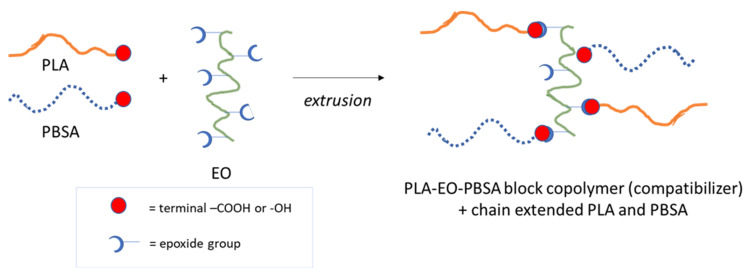
Simplified scheme of the reactions occurring in the melt between PLA, PBSA and EO.

In fact, the pure PLA showed a torque of 152.3 ± 3.0 N*cm and an MFR of 2.3 ± 0.3 g/10 min, but the pure PBSA was less viscous, showing a torque of 104.0 ± 7.1 N*cm and a MFR of 7.1 ± 0.7 g/10 min. Hence the fluidity in the melt and thus the processability of the produced granules can be modulated as a function of composition. This trend is in accordance with the necessity to appropriately modify the extrusion and injection molding for the different compositions, as explained in Section 2.2. Interestingly, the blends containing EO showed an increased value of torque and a significantly lower value of MFR, because of the increase in molecular weight due to the branching reactions of the EO. A simplified scheme of the reactions occurring in the melt between PLA, PBSA and EO is reported in Figure 2.

The lowest MFR value was recorded for the (80/20)J blend, thus the one that required an increase in the mold temperature and injection pressure during the injection molding process (as previously observed and reported in Table 2).

The main tensile properties (Young’s modulus, yield stress, stress at break, elongation at break and Charpy impact resistance) determined from the tensile stress–strain curves and Charpy impact test, are reported in Table 3.

**Figure 3:**
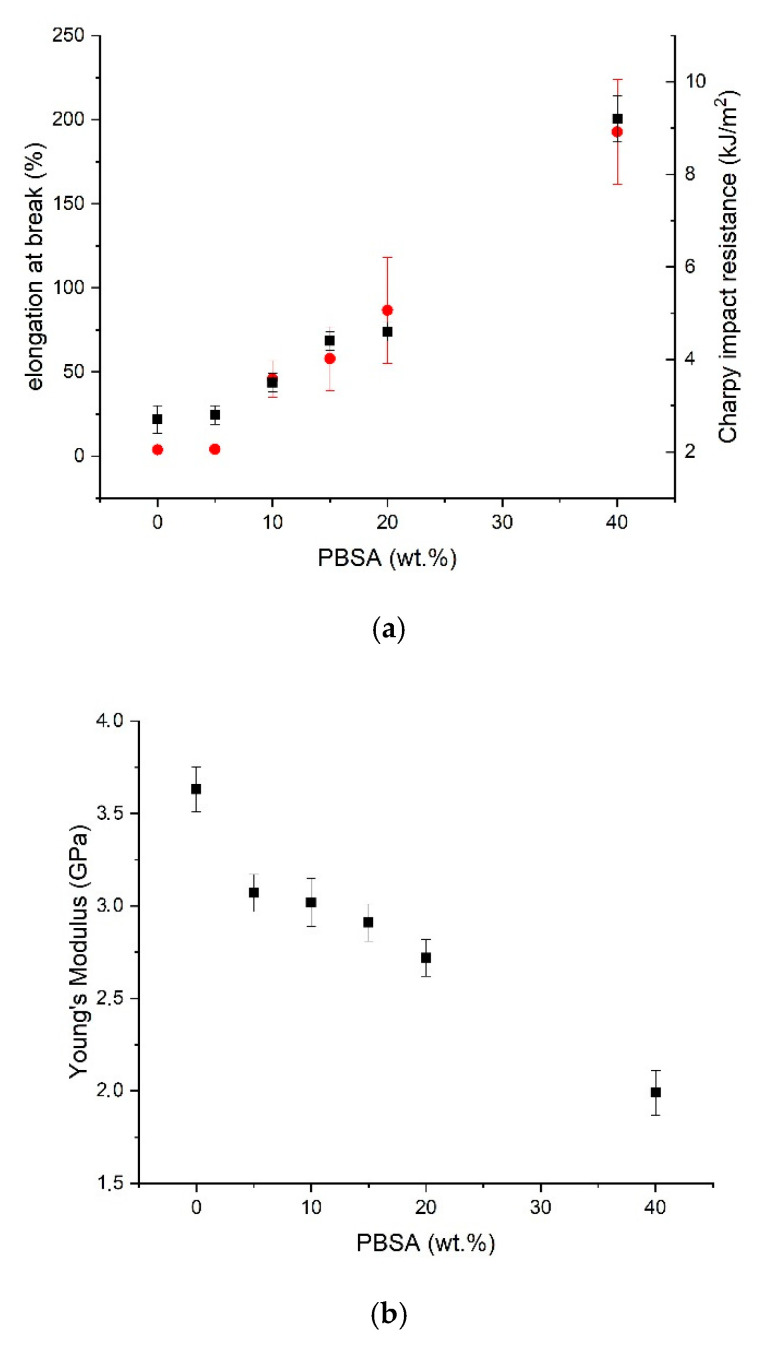
(**a**) Trend of elongation at break (red circles) and Charpy Impact strength (black squares) as a function of PBSA content; (**b**) trend of Young’s Modulus as a function of PBSA content.

The mechanical behavior of the blends changed, passing from neat PLA to binary PLA/PBSA blends. PLA alone had the typical mechanical response of a fragile material with a high stiffness and tensile strength but a low Charpy impact resistance and elongation at break. Neat PLA failed just after the elastic region (no yielding point is observed), as typical in a brittle fracture. The PBSA addition maintained the material stiffness at an acceptable level but at the same time improved its flexibility for contents higher than 5 wt.% of PBSA. In fact, 95–5 blend still showed a fragile behavior without yielding, and a low elongation at break, low Charpy impact resistance and still high stress at break. From 5 up to 40 wt.% of PBSA, the increment in elongation at break and Charpy impact resistance was almost proportional to PBSA content (Figure 3a). A marked decrement of stress at break was registered due to the elastic characteristic conferred by the growing PBSA addition. The variation of stress at break, also considering the values of the deviations reported, can be considered negligible. However, the slight stress at break increase for the 60–40 blend could be attributable to the change in the morphology of the blend which became co-continuous. Hence, also the more ductile PBSA phase results were continuous. Then, in this co-continuous blend both phases fully contributed to the blend mechanical response in all directions and resulted in a more effective stress transfer. 

The Young’s Modulus decreased by increasing the PBSA content in monotonic way (Figure 3b). Hence, in the investigated range a wide modulation of properties is possible by varying the blend composition.

**Figure 4:**
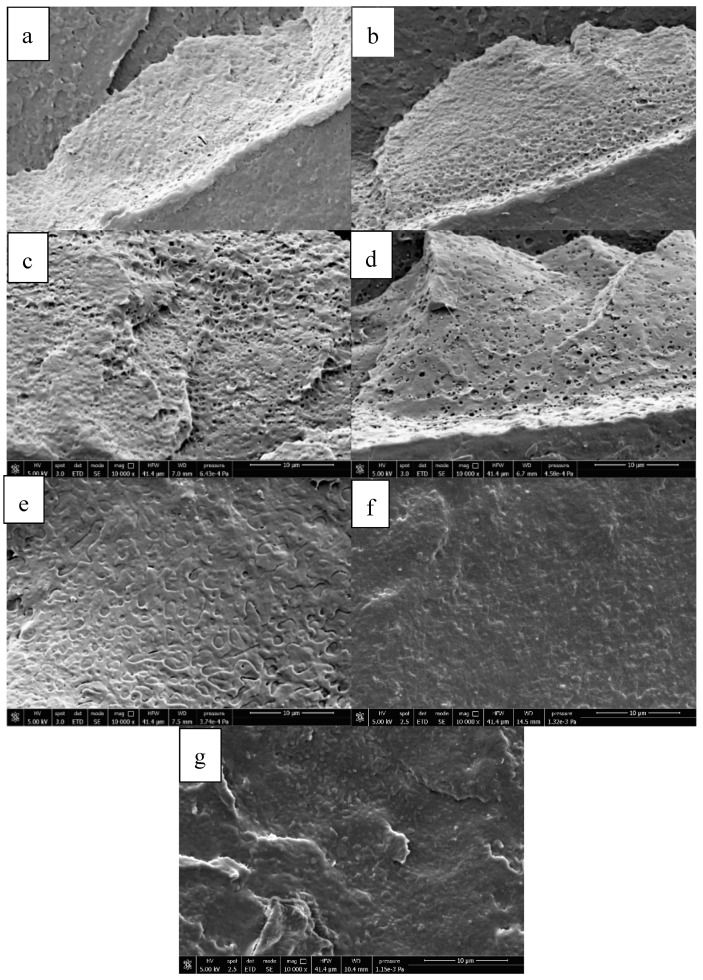
SEM micrographs of morphology phase development of PLA/PBSA blends: (**a**) 95–5; (**b**) 90–10; (**c**) 85–15; (**d**) 80–20; (**e**) 60–40; (**f**) (80/20)J; (**g**) (60/40)J.

The results of the mechanical tests are closely related to the materials morphology (Figure 4).

The PBSA, until 20 wt.% in the blends, appeared as a spherical/ellipsoidal dispersed phase in the PLA matrix, indicating the poor miscibility between PLA and PBSA. In some cases, it was possible to observe voids at the interface (debonding). The cause of this debonding could be related to the cryofractured samples, where dilatational stresses were generated to the mismatch of the thermal coefficients between the PBSA particles and the PLA matrix during cooling before fracture, as was also observed for PLA–PBAT system [20]. The 60–40 mixture confirmed what is generally observed in literature [38,41] where a change in morphology occurs (from PBSA dispersed particles to a co-continuous structure); so the remarkable improvement in elongation at break, especially of the impact strength observed, is explained with the achievement of the inversion point region.

A typical two-phase structure, where discrete droplets of the minor phase are dispersed in the matrix, was observed in the samples with PBSA content up to 20%. For these blends it was possible to calculate the *R_n_, R_v_* and *SD* values, given by the ratio between *R_v_* and *R_n_*, (reported in Table 4) according to Equations (5)–(7), respectively.

The average size of the domain was between 1 and 2 μm indicating that PBSA and PLA were thermodynamically incompatible [41]. A slight increment of *R_v_* values was observed with the increase in the PBSA content. This behavior is in agreement with the coalescence theory [73] for which, during the mixing process, the dispersed phase can collide with each other and coalesce, forming bigger droplets. The probability of droplet collision will be more accentuated by increasing the PBSA content, with the number of PBSA droplet collisions that will be proportional to the square of the PBSA concentration [74]. However, this size distribution increment is not as pronounced as could be expected. This behavior was encountered by other authors [70] and can be ascribed to the high shear rate and extensional flow during the extrusion process that limited the particle coalescence favoring the break-up of dispersed droplets. Moreover, the two polymers, being both polyesters, are characterized by a good chemical affinity in full agreement with the evolution of mechanical properties as a function of PBSA content. However, the dispersed phase dimensions are in accordance with the general rule regarding rubber toughening, which states that the dispersed phase must be distributed as small domains in the polymer matrix [75,76]. This aspect, combined with a sufficient interfacial adhesion, would increase the elongation at break and the impact properties of the final material in accordance with the results obtained from the mechanical tests.

The 60–40 blend showed a co-continuous morphology where the dispersed phase coalesced until it formed bigger structures throughout the whole blend [77,78,79,80]. Consequently, due to the irregularity of the shape assumed by this structure, the calculation of *R_v_, R_n_* and *SD* parameters was not feasible. The analysis of the micrographs has anyway shown that the dimension of the two interpenetrating phases is quite low, probably thanks to effective processing and this explain the possibility of a good modulation of properties by acting on composition. Moreover the presence of EO improved the compatibility of the phases: a decrease in phase dimensions can be noticed both in the (80/20)J and in (60/40)J blend (Figure 4f,g) as well as an increased interfacial adhesion [44,81,82,83] making difficult the observation of these interfaces during the analysis.

**Figure 5:**
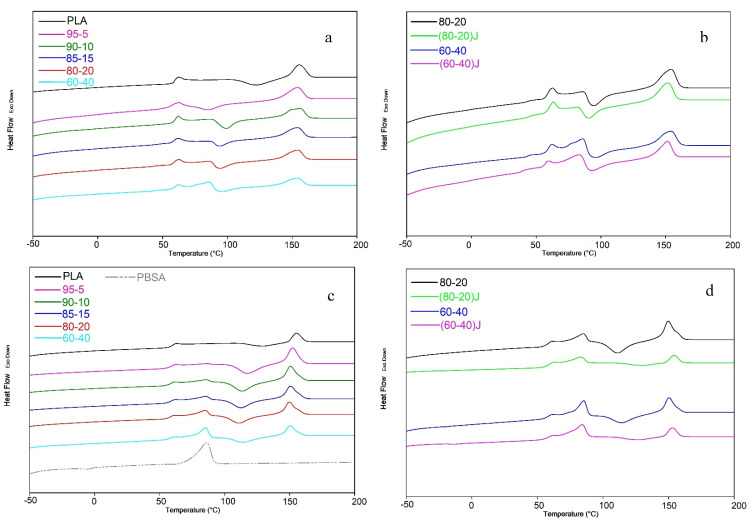
DSC thermograms (**a**) first heating for PLA/PBSA blends; (**b**) first heating for the selected blends with EO; (**c**) second heating for PLA/PBSA blends; (**d**) second heating for the selected blends with EO. Heat flow, reported in J/g, is expressed in arbitrary units to allow the curves shifting.

The first heating thermograms obtained from DSC analysis and reported in Figure 5a,b show the thermal history of the samples produced by injection molding.

The analysis of these data was preferred to get correlation with mechanical results, measured on injection molded specimens. Moreover, this analysis can provide useful information regarding the peculiar injection molding process adopted for the different blends. In fact, the crystallization occurring in the mold had a significant role in allowing a rapid and efficient ejection of the specimen by the machine without any distortion. Moreover, thermal properties related to the use of EO were yet investigated by Nunes et al. [84] in PLA. EO determined a decrease in the crystallinity in pure PLA and in blends with PBAT because of the introduced disorder due to the branching of chains. Lascano et al. [55] did not determine the crystallinity of their PLA/PBSA but the thermograms they reported were in good agreement with Nunes et al. [84] observations. These considerations can explain the observed necessity to increase the molding temperature in the injection molding of blends containing EO, to counterbalance the reduced tendency to crystallization with a decreased undercooling favoring the extension of the crystalline fraction.

According to literature [85,86], PBSA has a triple melting peak centered at around 87.14 °C; the melting behavior can occur with a melting peak numbers that depend on the processing conditions. Consequently, it was not possible to measure with accuracy the PBSA melting enthalpy and the PLA cold crystallization enthalpy due to the overlap between the PBSA melting endotherms, the PLA cold crystallization exotherms (that became more marked with increasing the PBSA content) and the enthalpic relaxation peak occurring in correspondence of the PLA glass transition temperature and ascribed to the specimen’s aging.

With the PBSA addition also the PLA double melting behavior started to become observable. These double peaks were correlated to the remelting of newly formed crystallite during heating. Crystallites of disordered α’ form (with low melting temperature) re-crystallize in a more ordered α form having a higher melting temperature. In any case, the right melting peak of PLA was deemed to be the one which was in a higher temperature range [10]. According to the melting recrystallization model [87] the small and imperfect crystals would be transformed into more stable and perfect crystals. However, at high cooling rates (like that reached during the injection molding process), the granules passed to the recrystallization region so fast that there was insufficient time for the molten material to reorganize into new ordered crystals generating consequently low melting crystallites [13]. In fact, it is known in literature that the heating rate influences markedly the conversion from α′-to α-form for PLA as well as the presence of PBSA double or triple- melting peaks [83,88,89]. The absence of PBSA cold crystallization in the blends and also in the pure material can be ascribed to different factors. The most important is probably correlated to the PBSA macromolecules having a very fast crystallization rate during cooling and this leading to an absence (or very low quantitative) of amorphous domains that could recrystallize again during heating [8].

For all the blends, the cold crystallization temperature of PLA was shifted to a lower temperature if compared to pure PLA (Figure 5a–d); in agreement with Lascano et al. [55], the PBSA melting contributes to the increase in the PLA chain motion allowing PLA chains to arrange into packed structures at lower temperatures. Consequently, the cold crystallization temperature and the melting temperature of PLA decreased with the PBSA addition. However, the most marked decrement of the PLA cold crystallization temperature was observed for the 95–5 blend (especially in the first heating scan, related to the injection molding cycle that could enhance the PBSA nucleating ability because of the high temperature of the mold and high holding time) where the PBSA dispersed phase, present in low content but with a low particle size, seemed to act as nucleating agent [10] thanks to the effect of the extended interface. In fact, in good agreement, by increasing the PBSA content, the PLA cold crystallization temperature rose slightly but still remained lower than that of pure PLA.

An enthalpic relaxation peak above the glass transition temperature due to the aging [90] could be observed in the first heating scan (Figure 5a,b) for all blends, but it was reduced in blends containing EO because of their more disordered structure hindering chain relaxation. However, for both heating scans, it could be observed that the glass transition temperature of PLA remained almost unchanged with PBSA content, confirming the restricted miscibility of the two biopolymers as reported by Lee et al. [13].

**Figure 6:**
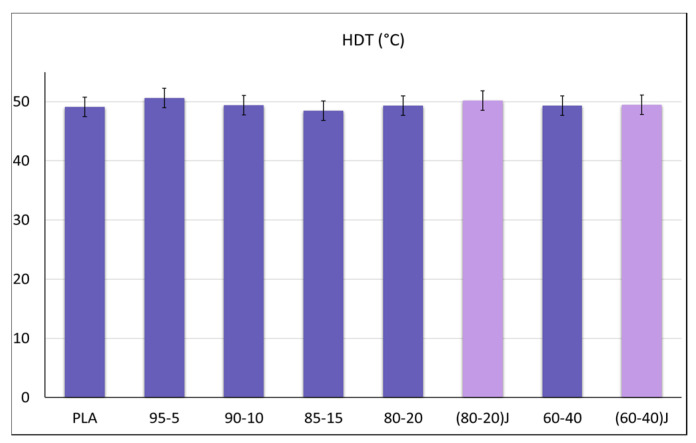
HDT values for all blend compositions.

In order to better understand the HDT obtained data (Figure 6), showing an almost constant value in all the examined blends, although it was not possible to determine the crystallinity values of the injection molded specimens considering the first heating scan, an estimation of the crystallinity was made on the second run. In this case, having deleted the thermal history, the single PBSA granules were also investigated. In fact, in the second run, thanks to the disappearance of the aging peak and to the lower cooling rate with respect to injection molding, the peaks of PBSA and PLA show an almost null overlap (Figure 5c,d).

**Figure 7:**
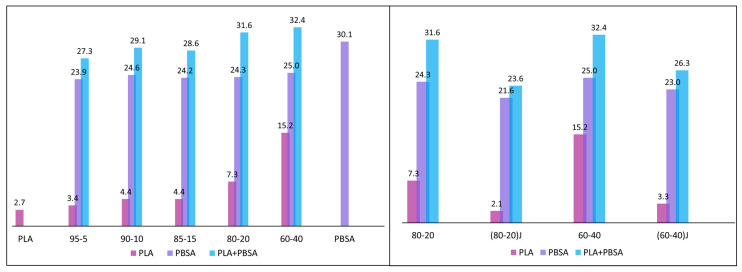
Crystallinity values for PLA/PBSA blends in the second heating scan.

In Figure 7 the crystallinity percentage values for PLA, PBSA and the sum of them (that is, the total crystallinity of the sample) are reported. The enhancement of PLA crystallinity with PBSA content, suggests that the PBSA droplets, dispersed into the PLA matrix effectively act as crystallization nuclei for PLA accelerating the crystallization during the heating process [10]. For all the blends the maximum total crystallization, seen as the sum of PLA and PBSA crystallinity percentage, was between 27 and 32%. Hence only small variations of the crystalline fraction were present in the different blends and this was reflected in the HDT values (Figure 6) which were approximately the same for all the examined blends. In fact, it is well known that the HDT of neat PLA is at about its *T*_g_. The HDT is significantly affected by the degree of crystallization [91]. The low crystallization rate of PLA made it essentially amorphous under the injection molding conditions adopted in this work. Although a quantitative evaluation of the samples crystallinity was not possible for all blend compositions, it could be supposed that the maximum degree of sample crystallinity was lower than that measured during the second heating run. Consequently, the low crystallinity (due to the low crystallization rate) reached by the PLA under practical molding conditions was reflected in a null variation of the HDT that remained almost unchanged for all the blends analyzed (Figure 6).

### 3.2. Effect of the EO on Mechanichal and Failure Behaviour of PLA/PBSA Blends

EO was added (at 2 wt.%) to 80–20 and 60–40 blends. These two formulations were selected because they showed the highest elongation at break and Charpy impact resistance values.

From a morphological point of view, it can be observed in Figure 3, that EO acted as a compatibilizer, working as a bridge between the PLA and PBSA phases, leading to a reduction of the interfacial tension and thus leading to better adhesion and dispersion between the two phases [55,92,93]. 

The interaction created by EO in the PLA–PBSA blends can also be observed in the torque values that for (80–20)J and (60–40)J were higher than the torque value of pure PLA (Figure 1). The MFR, on the other hand, reached the lowest values when EO was added. It is known that the torque increment is related to the molten polymer viscosity increase, which is caused by a molecular weight increment in polyesters [17]. Furthermore, the chain extender produces an increment of the melt viscosity creating interactions between the matrix and the dispersed phase. This marked viscosity variation with respect to the viscosity values recorded for corresponding blends without EO (80–20 and 60–40), led to a significant variation of the injection molding conditions (as can be observed in Table 2) where the mold temperature and the injection pressure were increased.

From a thermal point of view, it can be observed (Figure 5) that the EO addition caused a slight decrement of *T_g_* that could be ascribable to the increased compatibility between the two polymers [17]. A slight shift of the PLA cold crystallization temperature was also registered. On the other hand, the introduction of EO limited the chain mobility and the shift of PLA cold crystallization temperature did not occur in the presence of the chain extender. As could be expected, the chain extender depressed severely the PLA crystallization while the PBSA crystallization did not seem to be much influenced by the EO addition (Figure 7).

However, from the results of HDT (Figure 6), it could be detected that the EO addition did not cause a significant variation of the HDT values and it did not significantly affect the final crystallinity of the material. In any case the increase in molecular weight and branching activity of EO on PLA counterbalanced the decrease in crystallinity in this phase, thanks to the increased resistance of the amorphous phase achieved thanks to the formation of new inter-macromolecular bonds.

**Figure 8:**
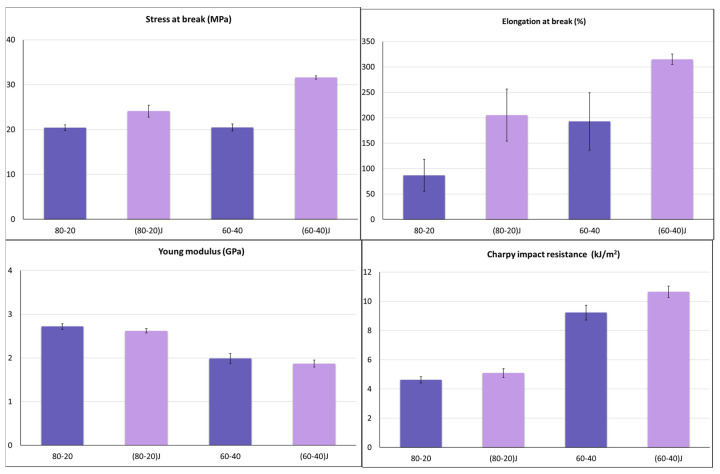
Comparison of the main mechanical properties for blends with and without Joncryl.

The enhanced compatibility induced by EO was overwhelmingly reflected in the mechanical properties (Figure 8). EO caused a relevant increment of the mechanical properties. Not only an improvement in elongation at break and Charpy impact resistance occurred, but also a marked increment in stress at break was recorded.

Essentially, the increase in stress at break and elongation at break may be related to the molecular weight increase, due to the polyester reaction with EO (Figure 2). On the other hand, the toughness increment is more related to the morphology of the system and, as can be expected, better toughness is reached for the (60–40)J blend where a co-continuous structure combined to a better phase compatibility was observed.

To better understand the effective toughness enhancement obtained with the EO addition, the *J_Ilim_* was calculated adopting the elasto-plastic fracture mechanism approach. The *J_Ilim_* values correspond to the energy absorbed by the specimen at the moment of the crack propagation in slow-rate test conditions. 

**Figure 9:**
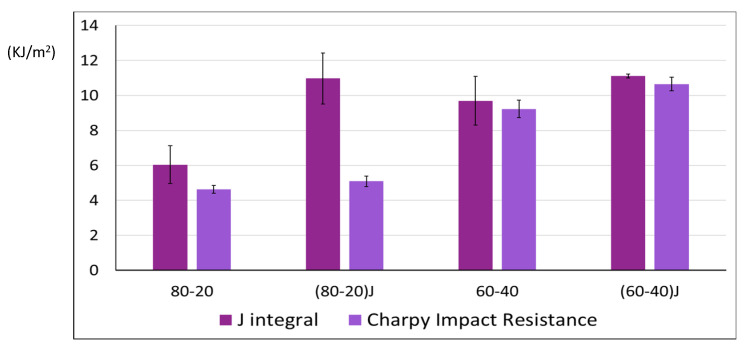
Comparison between J_Ilim_ and Charpy impact resistance values (both in KJ/m^2^) for blends with and without Joncryl.

Comparing the *J_Ilim_* values with the “brittle” *G* value of pure PLA (equal to 2.97 kJ/m^2^ [94,95]) all blends showed very interesting values (Figure 9). The fracture energy released at the beginning of crack propagation was markedly increased with the EO addition for the (80–20)J blend, while for the 60–40 composition the *J_Ilim_* value remained almost unchanged with the EO addition. Probably, the 60–40 blend being co-continuous, the effect of EO addition was less marked; the co-continuous nature of the blend led it to have a starting high value of *J_Ilim_* and Charpy impact resistance that were not significantly altered by the presence of EO.

**Figure 10:**
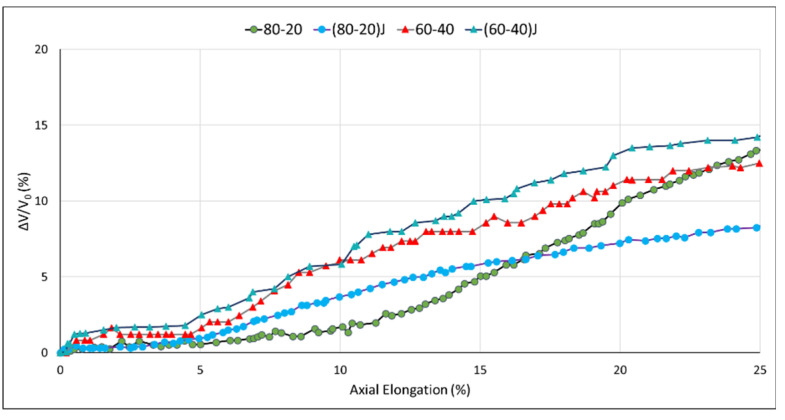
Volume strain–strain curves for blends with and without EO.

The results of dilatometric tensile tests are reported in Figure 10 where the volume variation (calculated by Equation (1)) is reported as function of axial elongation (the tests were carried out until the deformation of the specimens remained homogeneous).

The characterization of the dilatational response of a material when it is subjected to an applied stress can lead to an appreciated deformation mechanism in the bulk of the material. According to the type of volume strain–strain curve obtained, it is possible to distinguish between: a cavitational response, a dilational response and deviatoric response [96]. It can be observed that except for the 80–20 blend, where a cavitational response is observed, for the other blends a deviatoric response was registered. 

**Figure 11:**
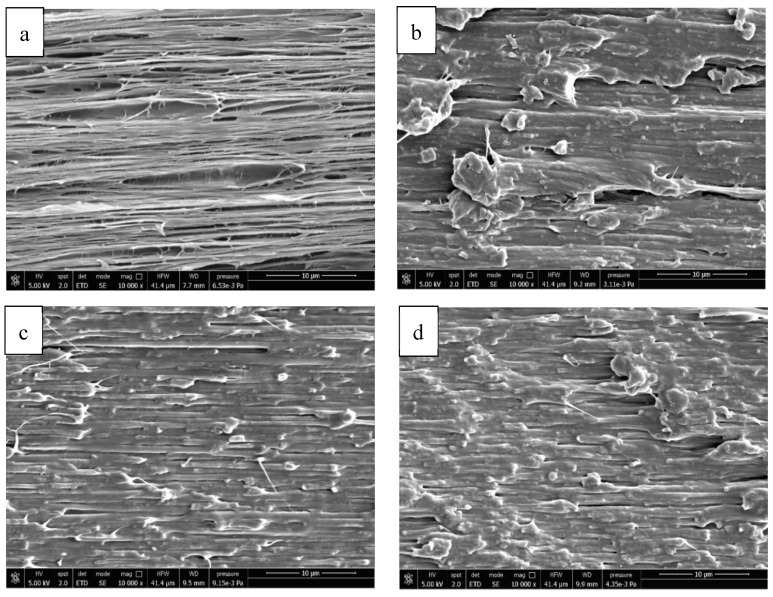
SEM micrographs made at the surface of tensile specimen cryo-fractured along the draw direction: (**a**) 80–20; (**b**) (80/20)J; (**c**) 60–40; (**d**) (60/40)J.

As a consequence, for the 80–20 blend, in the large deformation limit, the hydrostatic tensile stress will cause cavitation type mechanisms that will lead to a rapid volume increase. In particular, when the stress approaches the yield stress value, the cavitation-type mechanism will produce voiding that will cause the rapid increase in the volume strain, and the coalescence of these voids will bring to the final rupture of the specimen [60]. The SEM micrographs at the cryo-fractured surface of tensile specimens along the draw direction (Figure 11) confirmed the presence of many small and big elongated voids grown along the tensile direction due to cavitation for the 80–20 blend.

The volume dilatation response for the other blends ((80–20)J, 60–40 and (60–40)J)), displayed a deviatoric behavior; for these materials, when the stress value approaches stress at yield, the material will continue to deform by changing shape and not volume, leading to a shear yielding as the main failure mechanism. The SEM images confirmed that a different deformation mechanism occurred where the material underwent deformation along the draw direction without voiding formation.

Similar to what was observed for the *J_lim_* value, the EO addition in the 80–20 mixture, significantly changed the micromechanical behavior of the material. This marked change can probably be ascribed to the capacity of the chain extender to modify the macromolecule structure and create interactions between the matrix and the dispersed phase, modifying the initial interface adhesion and leading to a different micromechanical response. 

On the other hand, the effect of EO to the micromechanical behavior of the 60–40 blend could be considered negligible. The dilatometric curves of the 60–40 and (60–40)J blends were very similar with a very slight increment of the volume variation for the (60–40)J blend. In this case the micromechanical behavior was probably ascribable to the different morphology of the 60–40 blend that, being co-continuous, did not show dispersed PBSA particles into the PLA matrix. 

## 4. Conclusions

In this study, PLA/PBSA binary blends with different PBSA contents (from 5 up to 40 wt.%) were investigated from a rheological, thermal and mechanical point of view. To the blends (80–20 and 60–40), that presented a higher value of impact resistance and elongation at break, 2 wt.% of an epoxy oligomer (EO) as compatibilizer was added. The two blends also presented different morphologies: the 80–20 blend possesses a morphology where the PBSA particles are dispersed within the PLA matrix, while the 60–40 blend shows a co-continuous structure.

From the thermal properties point of view, no significant variations were observed caused by the EO addition, whereas, as could be expected, the interaction created by EO produced an increment of the melt viscosity.

However, the most interesting results were observed from the micro and macro mechanical analysis. The mechanical results showed that the Charpy impact resistance value (high speed test) and the *J_lim_* (energy absorbed from the specimen at the moment of the crack propagation in slow-rate test conditions) are higher for the 60–40 blends having a co-continuous morphology. However, for this 60/40 blend the EO addition does not significantly alter the mechanical response. In fact, dilatometric tests show that for the 60/40 blend the micromechanical response is of the deviatoric type and the morphology, investigated at the surface of cryo-fractured tensile specimen along the draw direction, was comparable. 

On the other hand, the 80/20 blend was significantly affected by the presence of EO. The micromechanical response passed from a cavitational response to a deviatoric one, probably induced by the marked change in the dispersed phase morphology with the EO addition and the higher complexity of macromolecule structure generated by the reactive processing. 

The systematic study described in this paper was thus useful for a better knowledge of thermo-mechanical behavior of PLA blends containing up to 40% of PBSA.

Moreover, the EO used in the present paper resulted in being quite useful in effectively modulating melt viscosity and this is necessary not only for adapting the material to several processing methodologies, but also as compatibility enhancer, impacting positively the mechanical performance. However, this additive is not biobased and biodegradable. Hence in the future it could be important to replace it with biobased and biodegradable ones, to grant a full circularity of the material by keeping into account both its origin and final disposal.

## Figures and Tables

**Table 1:** Blend names and composition.

Blend Name	PLA–PBSA wt.%	EO wt.%
PLA	100–0	0
95–5	95–5	0
90–10	90–10	0
85–15	85–15	0
80–20	80–20	0
(80–20)J	80–20	2
60–40	60–40	0
(60–40)J	60–40	2

**Table 2:** Injection molding conditions.

Main Injection Molding Parameters	PLA	95–5	90–10	85–15	80–20	(80–20)J	60–40	(60–40)J
Temperature profile (°C)	180/185/190							
Mold temperature (°C)	70	70	60	60	55	65	55	65
Injection holding time (s)	5							
Cooling time (s)	15				25			
Injection pressure (bar)	90	90	80	80	80	120	80	100

**Table 3:** Tensile and impact properties for each formulation containing an increasing PBSA content.

Blend Name	Young’s Modulus (GPa)	Yield Stress (MPa)	Stress at Break (%)	Elongation at Break (%)	Charpy Impact Resistance (kJ/m^2^)
PLA	3.63 ± 0.12	/	62.7 ± 1.7	3.7 ± 0.3	2.7 ± 0.3
95–5	3.07 ± 0.1	/	59.4 ± 0.1	4.0 ± 0.2	2.8 ± 0.3
90–10	3.02 ± 0.13	57.4 ± 0.7	23.3 ± 2.3	45.8 ± 10.9	3.5 ± 0.2
85–15	2.91 ± 0.10	55.3 ± 0.7	21.3 ± 1.1	57.9 ± 18.9	4.4 ± 0.2
80–20	2.72 ± 0.10	51.2 ± 0.6	19.7 ± 0.6	86.7 ± 31.5	4.6 ± 0.2
60–40	1.99 ± 0.12	39.9 ± 0.8	21.5 ± 0.8	192.8 ± 31.1	9.2 ± 0.5

**Table 4:** *R_n_*, *R_v_* and *SD* values of the dispersed PBSA phase for PLA/PBSA blends at different compositions up to 20 wt.% of PBSA content.

Blend Name	*Rn* (μm)	*Rv* (μm)	*SD*
95–5	0.41	0.54	1.32
90–10	0.42	0.58	1.38
85–15	0.54	0.78	1.44
80–20	0.55	0.80	1.45

## Data Availability

The data presented in this study are available on request from the corresponding author.
